# Group antenatal care versus standard antenatal care and effect on mean gestational age at birth in Rwanda: protocol for a cluster randomized controlled trial

**DOI:** 10.12688/gatesopenres.13053.1

**Published:** 2019-09-27

**Authors:** Sabine Furere Musange, Elizabeth Butrick, Tiffany Lundeen, Nicole Santos, Hana Azman Firdaus, Alejandra Benitez, David Nzeyimana, Nathalie Kayiramirwa Murindahabi, Lauriane Nyiraneza, Felix Sayinzoga, Vedaste Ndahindwa, Fidele Ngabo, Jeanine Condo, Dylis Walker

**Affiliations:** 1 School of Public Health, College of Medicine and Health Sciences, University of Rwanda, Kigali, Rwanda; 2 Institute for Global Health Sciences, University of California San Francisco, San Francisco, USA; 3 University of California, Berkeley, Berkeley, USA; 4 Maternal, Child and Community Health Division - Institute of HIV/AIDs, Disease Prevention and Control, Rwanda Biomedical Center, Kigali, Rwanda; 5 Rwanda Biomedica Center, Kigali, Rwanda; 6 Obstetrics, Gynecology and Reproductive Sciences, University of California San Francisco, San Francisco, USA

**Keywords:** Antenatal care, Postnatal care; Group care, Group based antenatal care, Group prenatal care, Centering Pregnancy, Sub-Saharan Africa, Pregnancy, Preterm birth, Gestational age

## Abstract

**Background:** Group antenatal care has demonstrated promise as a service delivery model that may result in improved outcomes compared to standard antenatal care in socio-demographic populations at disparately high risk for poor perinatal outcomes. Intrigued by results from the United States showing lower preterm birth rates among high-risk women who participate in group antenatal care, partners working together as the Preterm Birth Initiative - Rwanda designed a trial to assess the impact of group antenatal care on gestational age at birth.

**Methods:** This study is a pair-matched cluster randomized controlled trial with four arms. Pairs randomized to group or standard care were further matched with other pairs into quadruples, within which one pair was assigned to implement basic obstetric ultrasound at the health center and early pregnancy testing at the community. At facilities randomized to group care, this will follow the opt-out model of service delivery and individual visits will always be available for those who need or prefer them. The primary outcome of interest is mean gestational age at birth among women who presented for antenatal care before 24 completed weeks of pregnancy and attended more than one antenatal care visit. Secondary outcomes of interest include attendance at antenatal and postnatal care, preterm birth rates, satisfaction of mothers and providers, and feasibility. A convenience sample of women will be recruited to participate in a longitudinal survey in which they will report such indicators as self-reported health-related behaviors and depressive symptoms. Providers will be surveyed about satisfaction and stress.

**Discussion:** This is the largest cluster randomized controlled trial of group antenatal and postnatal care ever conducted, and the first in a low- or middle-income country to examine the effect of this model on gestational age at birth.

**Trial registration:** This study is registered on ClinicalTrials.gov as NCT03154177 May 16, 2017.

## Abbreviations

ANC, antenatal care, CHW, community health worker; CONSORT, Consolidated Standards of Reporting Trials; CRCT, cluster randomized controlled trial; DSMB, Data Safety and Monitoring Board; EDD, estimated date of delivery; GA, gestational age; LMIC, low- and middle-income country; MOH, Ministry of Health (Rwanda); PNC, postnatal care; PTB, preterm birth; PTBi – Rwanda, Preterm Birth Initiative – Rwanda; RBC, Rwanda Biomedical Center; RCT, Randomized controlled trial; REDCap, Research Electronic Data Capture; SPIRIT, Standard Protocol Items: Recommendations for Intervention Trials; UCSF, University of California, San Francisco; UPT, Urine pregnancy test; UR, University of Rwanda; WHO, World Health Organization.

## Introduction

Group antenatal care has demonstrated promise as a service delivery model that may be superior to standard antenatal care in socio-demographic populations at disparately high risk for poor perinatal outcomes, with no report of harms. Well-designed randomized controlled trials (RCTs) demonstrating improved outcomes among women and newborns after participation in group antenatal care (ANC) compared to standard ANC have been conducted in the United States ^1– 6^ and Sweden ^7^, which are classified as ‘high-income economies’ and Iran ^8, 9^, which is classified as an ‘upper middle-income economy ^10^.’ Benefits reported in these trials, at statistical significance, include greater gestational age (GA) at birth, greater birth weight, lower incidence of sexually transmitted infections, healthier maternal weight trajectories, fewer depressive symptoms, and increased satisfaction with care.

Among low-income countries, several small studies have reported on group ANC feasibility, acceptability, and prospective cohort outcomes. Only one has reported results of a small individual RCT. A prospective cohort trial in Ghana reported significantly higher health literacy among women who participated in group ANC ^11^. A pilot study in Tanzania and Malawi reported feasibility and acceptability and a significant increase in attendance at five ANC visits among women randomized to group ANC ^12, 13^. A cluster RCT currently underway in Bangladesh will report on ANC and postanatal care (PNC) service coverage, skilled birth attendance, and institutional deliveries ^14^, and this group has already reported start-up and implementation costs of group ANC delivery in that context, including the average cost per participant ^15^.

Given these studies and ongoing RCTs, implementation and policy teams around the world would like to know if group ANC will deliver significantly improved outcomes among women living in low-income economies, which bear the highest burden of maternal and neonatal mortality. Furthermore, the World Health Organization (WHO) encourages the provision of group ANC “by qualified health professionals . . . in the context of rigorous research, depending on a woman’s preferences . . .” ^16^ This call for research underscores the timeliness of such studies.

The Preterm Birth Initiative (PTBi) – Rwanda, aims to explore both the outcomes and feasibility of a combined group ANC and group PNC model implemented in Rwanda. PTBi – Rwanda is a partnership between investigators at the University of Rwanda (UR) and University of California, San Francisco (UCSF) and national health system implementors at the Rwanda Biomedical Center (RBC) and Ministry of Health (MOH). PTBi - Rwanda’s primary aim is to reduce the burden of morbidity and mortality related to prematurity. Intrigued by results from the United States showing lower preterm birth (PTB) rates among high-risk women who participate in group ANC, these partners aim to test the hypothesis that Rwandan women receiving care at facilities that offer group ANC will have a greater GA at birth, on average, than women receiving care at facilities that offer standard ANC. In addition, partners felt an important benefit of the approach was the ability to improve adherence to recommended ANC and PNC schedules, and also considered that additional components might produce similar effects. Specifically, partners wanted to study the effects of introducing urine pregnancy testing by community health workers as part of pregnancy surveillance and the use of ultrasound at first trimester to determine GA and motivate mothers to attend ANC earlier and more often.

Rwanda’s national maternity care system provides an excellent opportunity to test this innovative service delivery model due to its well-developed community capacity, cultural foundations in community-based decision-making and cooperation, and robust existing data system such as longitudinal ANC registers. The Rwanda MOH currently recommends four focused ANC visits and four PNC visits at 24 hours, 2 days, 3-7 days, and 42 days of life. The Rwanda Demographic and Health Survey published in 2015 provided a snapshot of the contemporary utilization of perinatal services ^17^. In Rwanda, 99% of pregnant women have attended at least one ANC visit with skilled medical personnel, while only 44% have attended the recommended four routine ANC visits. Of all births, 91% occur in a health care facility. While GA estimates are problematic as early ultrasound is not routinely available, 56% of women are reported to enter ANC before 16 weeks of pregnancy. About 19% of newborns receive a PNC assessment in the first two days after birth, but the proportion of women and newborns who receive PNC at about 42 days after birth has not yet been reported.

This article describes the design and intended evaluation of PTBi - Rwanda’s cluster RCT (CRCT) of a combined group ANC/PNC model, including primary and secondary outcomes, study population and sites, data collection methods, and data analysis plans.

## Methods

### Study aims

The primary aim of this study is to evaluate the effect of group antenatal care on gestational age at birth, at a cluster level. The secondary aims of this study are to determine the effects of: group antenatal care, screening obstetric ultrasound, and urine pregnancy testing in the community on antenatal care attendance and women’s and providers’ experience of antenatal and postnatal care; group antenatal care on preterm birth rates; group antenatal care on mode of birth; and group antenatal care on the outcomes of neonates at 42 days after birth, specifically among preterm neonates.

### Study design

The study is a pair-matched CRCT with four arms. In the five Rwandan districts selected for this study, we assessed 55 health centers to inform study design and site selection. First, we limited the data set to only those with an average of at least 48 first ANC per month, operating on the assumption that this would allow for formation of at least 2-4 ANC groups in the event the health center was allocated to the intervention. This gave us 50 potential health centers. We then limited to those that had more than one ANC provider during ANC days, which reduced the available health centers to 37. In total, 36 health centers (one was not matched to ensure pairing) were selected to participate in the study (see *Extended data*: File 15) ^18^.

Next data on all other criteria for each health center was used to perform nonbipartite pair matching in R producing a list of strongest potential matches across all criteria. The study team then reviewed the possible matches and agreed on optimal pairings among facilities that had at least 2 ANC providers prioritizing matches with similar volume of ANC1 visits, and similar distance to the nearest district hospital. Within each pair, one facility was randomized to group ANC and PNC using a computer-generated random assignment and the other was randomized to continue delivering the standard models of focused ANC and PNC. Selected health centers were pair-matched based on 1) number of ANC providers, 2) ANC volume 3) delivery volume, 4) proportion of ANC1 before 16 weeks gestation, 5) baseline PTB rate, and 6) availability of key screening tests. Pairs were then further matched into quadruples by the study team based on ANC volume, where one pair within each quadruple was randomly assigned using a computer-generated random assignment to additionally implement basic obstetric ultrasound at the health center and early pregnancy testing at the community level and the other was not. No allocation concealment was used.

**Figure 1.**
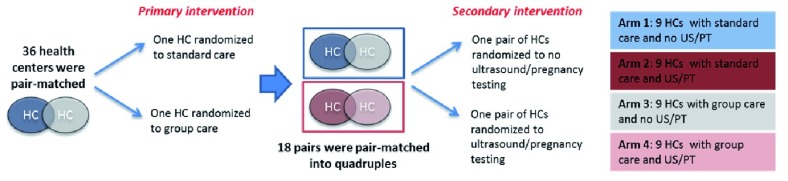
Facility-level randomization of 36 health centers included in a cluster randomized controlled trial of group antenatal care and postnatal care in Rwanda. Arm 1 delivers standard antenatal and postnatal care only and is the pure control. Arm 2 delivers standard antenatal and postnatal care, with the addition of early pregnancy testing in the community and obstetric ultrasound performed by primary antenatal care providers. Arm 3 delivers group antenatal and postnatal care, and Arm 4 delivers group antenatal and postnatal care with the addition of early pregnancy testing and ultrasound. In health centers randomized to group care (Arms 3 and 4), this model will follow the opt-out standard of care for facility-based antenatal and postnatal care. This design makes it possible to analyze differences between group care and standard care and also to assess if those effects are mediated by early pregnancy testing in the community or the availability of ultrasound (especially for early gestational age assessment) at the health center. HC, health center; US, ultrasound; PT, pregnancy testing.

Therefore, each study facility has one of the four assignments as illustrated in Figure 1. Arm 1 delivers standard ANC/PNC care only and is the pure control. Arm 2 delivers standard ANC and PNC care, with the addition of early pregnancy testing in the community and obstetric ultrasound performed by primary ANC providers. Arm 3 delivers group ANC/PNC, and Arm 4 delivers group ANC/PNC with the addition of early pregnancy testing and ultrasound. In health centers randomized to group care (Arms 3 and 4), this model will follow the opt-out standard of care for facility-based ANC and PNC. This design makes it possible to analyze differences between group care and standard care and also to assess if those effects are mediated by early pregnancy testing in the community or the availability of ultrasound (especially for early GA assessment) at the health center.

### Study setting

**Figure 2.**
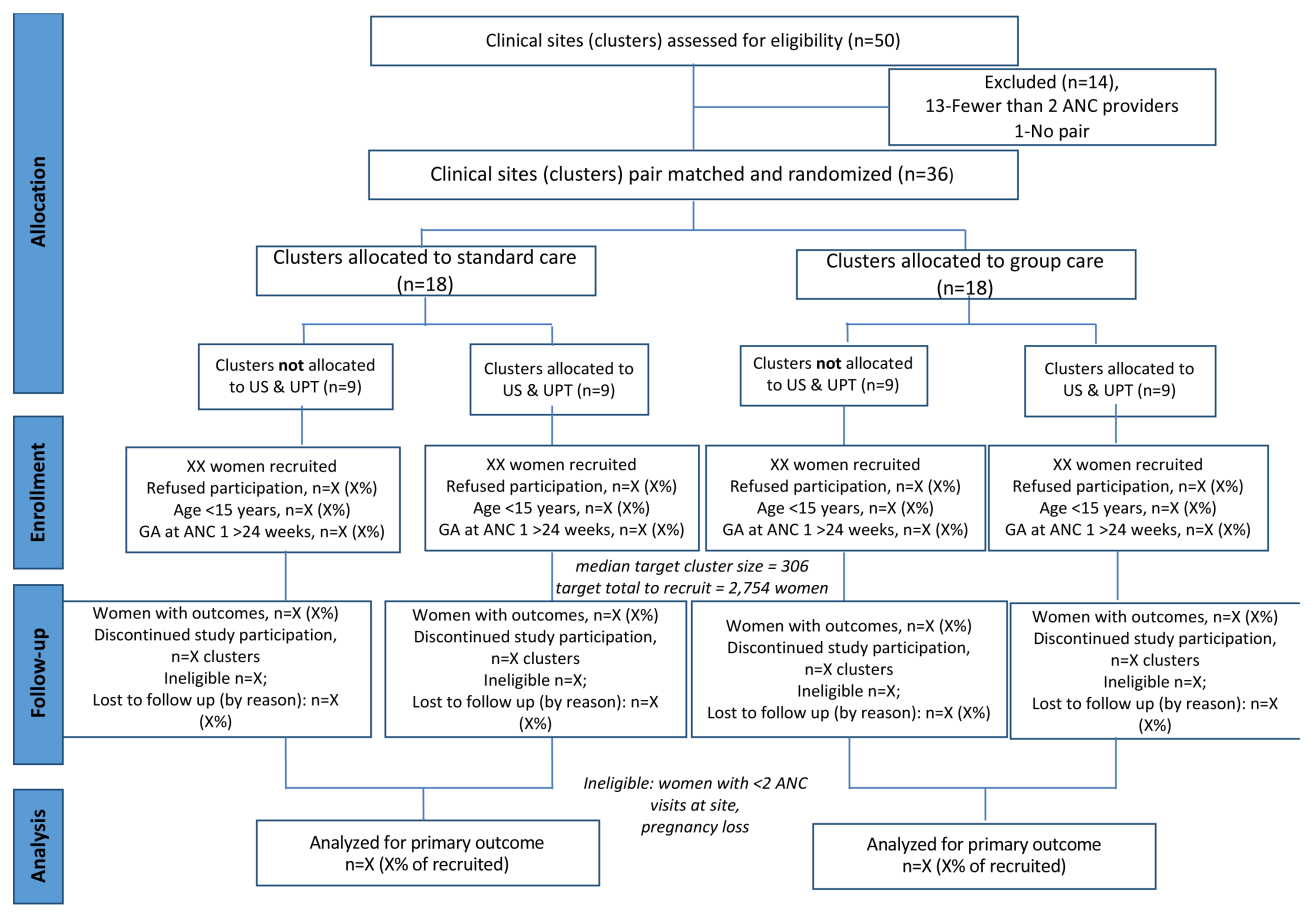
CONSORT flow diagram ^19^. ANC, antenatal care; US, ultrasound; UPT, urine pregnancy test; GA, gestational age.

This trial will evaluate the impact of a customized group ANC/PNC model in selected health centers in the five districts with the highest rates of preterm birth recorded in the health management and information system in Rwanda and with no overlapping project in place (Bugesera, Rubavu, Nyamasheke, Nyarugenge, and Burera). Health centers are the first facility point of contact for clients and provide a minimum package of services including promotional, preventive and curative care. At health centers, nurses and midwives offer universal access to ANC and PNC. Thirty-six health centers were selected for this CRCT based on their location in one of five districts, their average monthly ANC volume, and the reported presence of more than one ANC provider at the facility on any day ANC is offered there. All of the invited health centers agreed to participate. Study sites include urban and rural settings. Outcome data is also collected from District Hospitals to which these health centers refer in the event of complications. Figure 2 illustrates the trial design as a Consolidated Standards of Reporting Trials (CONSORT) flow diagram ^19^ following the Standard Protocol Items: Recommendations for Interventional Trials (SPIRIT) guidelines (see *Reporting guidelines*) ^18, 20^.

### Participant characteristics

Study participants include pregnant women and health care providers working in the participating health facilities. Pregnant women meeting the following criteria will be included in the primary analysis comparing the effect of group ANC versus standard ANC on gestational age at birth:

– Minimum age of 15 years at the time of enrollment
– Attend ANC1 before 24 completed weeks of pregnancy
– Attend more than one ANC visit at one of the 36 study facilities
– Consent to participate in the study and follow up

Women who present for ANC after 24 completed weeks of pregnancy will be invited to participate in group ANC throughout the remainder of their pregnancies, and descriptive data of their attendance will be reported; however, their outcomes will not be included in the primary analysis.

Women who only attend one ANC visit at a study facility will be excluded from the primary analysis. Health care providers working in selected health facilities and providing ANC services who are willing to participate will be included in this study.

### Study interventions

The desire to implement group ANC in this setting required the reconsideration or customization of an intervention that has been well-defined in the context of trials in high-income economies. In most trials of group ANC, the intervention is the trademarked product CenteringPregnancy® ^21^, but for three reasons we did not assume that the existing intervention could be applied ‘off-the-shelf” in Rwanda. First, the number of visits in the CenteringPregnancy® package is greater than the number of visits recommended in the Rwanda ANC package; second, the last Demographic and Health Survey estimated that one-third of Rwandan women could not read or write well; ^17^ and finally, the unique language (Kinyarwanda) and cultural context called for customized discussion activities.

The Rwanda group ANC/PNC model was customized by the Technical Working Group, which is composed of representatives from maternal-child health stakeholder organizations in Rwanda; that model development process is described separately ^22^. Key characteristics of the Rwanda group ANC/PNC model appear in Box 1. Despite the 2016 WHO ANC recommendations ^16^, the total number of ANC visits recommended in Rwanda is four. Of the four total PNC visits recommended in Rwanda, only PNC 4 (at approximately six weeks after birth) is a group visit. The other PNC visits do not lend themselves to a group arrangement and remain unchanged. PNC 1 is completed before facility discharge within 24 hours after delivery, and PNC visits 2 and 3 are conducted at home by community health workers.

Box 1. **Key characteristics of group antenatal care (ANC) and postnatal care (PNC) model.**

1. Women sit in a circle in a group space where other staff and patients do not enter during the visit.
2. Two co-facilitators lead each group visit: one ANC provider (midwife or nurse) and one Community Health Worker (CHW) with special training in maternal-child health.
3. Confidentiality and mutual respect are prioritized by pregnant women and co-facilitators.
4. Clean water is offered to the women to drink while they socialize during the first 30 minutes.
5. Health assessments are conducted on a rolling basis during the first 30 minutes, as women arrive at the scheduled visit time.
6. Women participate in their own health assessments as much as possible (blood pressure and weight measurements).
7. Brief consultations, including assessments such as fundal height measurement, are conducted in a semi-private area of the group space.
8. Women and newborns receive the routine assessments, screening, and treatments described in the Rwanda ANC/PNC packages, as well as treatments indicated for special conditions.
9. Women are referred to the district hospital for abnormal conditions, according to current national guidelines. The doctor with whom a referred woman consults will develop a plan of care and indicate on the counter-referral form whether or not she should continue to attend ANC visits at the health facility.
10. Group discussion begins after health assessments are completed and lasts 1 hour.
11. Key messages consistent with Rwanda’s ANC and PNC packages are delivered through facilitated discussion.
12. Learning activities are based on principles of adult education, including repetition, peer-to-peer teaching, and fun.
13. Each group of women decides if they will invite husbands and next-of-kin to attend group visits.
14. Co-facilitators “debrief” after every group visit in a continuous learning and quality improvement process.
15. Women are invited to return to the health facility at any time for individual episodic evaluation of danger signs or any other concerns.

Group ANC and PNC visits are timed according to the schedule in Table 1. This schedule places all group care visits eight weeks apart and simplifies group scheduling for the health facility. Those attending the first ANC visit in late in pregnancy are invited to attend the remaining scheduled visits of their assigned group (by estimated date of delivery, known as EDD) and are also invited to attend the visits they have missed with different groups as a ‘drop-in’ guest. It is ideal for a group of women to be entirely consistent over time; however, it is practical to plan for some movement of women between groups as needed. The goal is that by six weeks after birth women will meet for a final group visit with other mothers they already know and with whom they have formed close connections during group ANC.

**Table 1.** Rwanda group antenatal care (ANC)/group postnatal care (PNC): visit timing and curriculum content.

Visit	Timing	Educational Content
ANC 1 (standard, one-on-one initial pregnancy visit)	Variable: ideal is before 16 weeks gestation	Standard (e.g. HIV counseling and testing) Introduction to group care model and invitation to participate
ANC 2 (1st group visit)	20–24 weeks	Nutrition, supplements, and harmful substances Pregnancy danger signs Infection prevention and treatment
ANC 3 (2 ^nd^ group visit)	28–32 weeks	Birth plan (includes signs of labor) Healthy birth spacing and family planning Maternal mental health Review pregnancy danger signs
ANC 4 (3 ^rd^ group visit)	36–40 weeks	Respectful maternity care Breastfeeding and newborn care Postnatal and newborn danger signs Review family planning Review pregnancy danger signs
PNC (4 ^th^ group visit)	Approximately 6 weeks after birth	Review breastfeeding and infant feeding Review newborn danger signs Preventing health problems (e.g. insecticide-treated nets, hygiene, immunizations) Newborn and infant cognitive development (sing, talk, read, play)

When a pregnant woman presents at any health facility randomized to either Arm 3 or Arm 4 for her first ANC visit (ANC 1), she will experience a standard individual visit with a provider. At the conclusion of the ANC 1 visit, if ongoing ANC at the health center is most appropriate for the pregnant mother, the provider will invite the woman to participate in group ANC. Individual visits will continue to be offered for women and newborns requiring episodic examination and management, and for those who may decline group care participation. After the ANC provider identifies the woman’s due date based on best obstetric estimate, a member of the study staff assigns the woman to a group of 8–12 women with similar due dates—within the same two-week period is preferred and within the same four-week period is acceptable. Once the woman has been assigned to a group, all the dates of her group ANC visits 2–4 and group PNC visit at six weeks are known and the study staff communicate these to her either all at once or one at a time (at each visit), based on the provider’s preference. All women enrolled in the trial—both at control and intervention sites—will be encouraged to give birth in a facility, where the first PNC visit is delivered, to seek PNC care from the community health workers (CHWs) when the newborn is about three days and seven days old, and to return to the facility for PNC when the newborn is about six weeks old.

In Rwanda, ANC and PNC services are provided at each health center by advanced diploma-level nurses; a minority of these nurses have qualified as midwives. If physician care is required, women are referred to the district hospital for advanced services. A robust network of CHWs with special training in and responsibility for maternal and newborn health have frequent village-level contact with pregnant women. These two groups of health care workers were selected to function as co-facilitators (one ANC/PNC provider and one CHW) of each group visit.

Those providers and CHWs associated with sites randomized to group ANC were invited to a three-day training workshop on a rotating basis to minimize the impact on service provision. This workshop brought providers and CHWs together in joint training activities for the first time ever. Training occurred in large- and small-group circles to replicate as closely as possible the principles of successful group care. Activities focused on principles of adult learning, facilitation skills, a review of the Rwanda ANC/PNC packages of care, health assessment skills, how to organize and facilitate a group visit, and role-play practice of each of the group ANC/PNC curriculum discussion topics with feedback and re-practice.

These new group ANC facilitators were trained in all aspects of model fidelity by a team of six Rwandan group ANC Master Trainers ^22^. The Master Trainers, five midwives and one physician, who all have separate employment, were recruited based on their excellent facilitation skills, expertise in maternal and newborn health, and commitment to the project. The Master Trainers will make scheduled visits to each health center providing group ANC, with decreasing frequency over time, and unscheduled visits will be made as needed. During these visits, Master Trainers will observe group ANC as it is delivered and collect data about model fidelity. They will also offer supportive feedback and will model for group ANC facilitators how to plan for and debrief after each visit.

Study sites assigned to either Arm 2 or Arm 4 are randomized to introduce basic obstetric ultrasound by ANC providers at the health center and urine pregnancy testing by CHWs in the community ( Figure 1). The objective of screening ultrasound at the health center level is to more accurately assign the EDD, but providers are also trained to screen for gross abnormalities. The goal of community-based pregnancy testing is to engage women in earlier ANC attendance, which in turn may identify and promote early referral of at-risk pregnancies for physician consultation at district hospitals.

Members of the Rwanda Society of Radiologists developed training materials, provided training in basic obstetric ultrasound examination, and defined a mentorship and quality assurance plan. Two to three ANC providers (nurses & midwives) from each health center in Arms 2 and 4 and one radiography technician from each of the six district hospitals attended a 10-day obstetric ultrasound course created for frontline health workers customized for this context. The hospital-based radiography technicians were invited to serve as on-site ultrasound mentors to the ANC providers at the health centers associated with each hospital catchment area. They will provide frequent on-site mentorship during the first months of ultrasound implementation and then conduct monthly mentorship visits through the remainder of the study period. Members of the Society of Radiologists will engage in quarterly onsite visits at health centers and will conduct quality control by remotely reviewing securely shared files that include US examinations and their reports. Women who seek ANC at health centers in Arms 2 and 4 are offered a basic screening obstetric ultrasound either on the day of their first ANC visit or soon after.

Over 700 CHWs assigned to support maternal and newborn care in the catchment areas of health centers in Arms 2 and 4 were trained to administer a projected 25,000 urine pregnancy tests (UPTs) per year. The UPT training curriculum was developed in Kinyarwanda and English by RBC, which will also procure and distribute UPT kits and gloves. Women who test positive for pregnancy in the community are referred to the health center for early ANC. The CHW supervisor associated with each health center will provide ongoing monitoring and supervision of community-based UPT activities and report to the health center on a monthly basis.

Participation in any of these three interventions—group care, obstetric ultrasound, and urine pregnancy testing—is voluntary for any potential trial participant. All women who present for the first ANC visit are invited to consent to participate in the trial, in any of the four arms. Women who have consented to participate in the trial can withdraw their consent at any time for any reason.

### Outcomes and processes for comparison

This trial will evaluate both outcomes and processes. The primary study outcome is GA at birth. GA at birth will be calculated using the EDD assigned by ANC providers using the best obstetric estimate. In health centers randomized to implement basic obstetric ultrasound, this tool will be used to optimize GA assignment. In addition, postnatal measurements of every newborn will be used to estimate GA at birth and assign a corrected GA if needed.

Secondary outcomes include attendance, PTB rate, and neonatal mortality and morbidity among preterm neonates. Outcomes, including analysis metrics, method of aggregation and time points are summarized in Table 2.

**Table 2.** Outcomes.

Measurements variable	Analysis metric	Aggregation	Time Point
Primary Outcomes			
GA at birth	Average weeks completed	Health center	At delivery
Secondary Outcomes			
Preterm birth	Yes/no <37 weeks GA	Rate compared across arms	At delivery
Mortality among preterm neonates	Alive/dead	Rate compared across arms	At 28 days and 42 days, measured at six week follow up visit
Attendance at four ANC visits	Yes/no	Proportion compared across arms	At Delivery
Attendance at ANC one <14 weeks GA	Yes/no	Proportion compared across arms	At ANC 1
Attendance at PNC at six weeks	Yes/No	Proportion compared across arms	Record review at 12 weeks post EDD
Women identified as being high risk at ANC	Yes/no	Proportion compared across arms	From 1 ^st^ ANC until day of delivery
Caesarean section	Yes/no	Proportion compared across arms	At delivery
Newborn morbidities: jaundice, rapid breathing, fever, pneumonia, hypothermia, cord infection	Any reported: Yes/no	Proportion compared across arms	Reported in Rapid SMS (community reporting system) or neonatal register by 28 days of age
Other Outcomes			
Acceptability	Qualitative data on acceptability to women and providers	Qualitative reporting	At nine and 18 months
Satisfaction with care, locus of control, perceived social support, perceived stress, and depressive symptoms	Survey data	Comparison across arms	Baseline (at enrollment) and eight weeks after birth
Postnatal health behaviors, including family planning uptake, insecticide- treated mosquito net use, and breastfeeding	Survey data	Comparison across arms	Eight weeks after birth

GA, gestational age; ANC, antenatal care; PNC, postnatal care; EDD, estimated date of delivery.

We will also report several descriptive results of interest and identify factors that influence uptake, fidelity, and sustainability of group care in Rwanda. This includes the proportion of ANC and PNC visits at sites randomized to group care that occur in a group versus one-on-one with a provider. We will describe attendance at group care visits by men or female ‘next of kin’. Uptake will be measured by enrollment and mothers’ attendance at scheduled group antenatal care (gANC) visits. Furthermore, a subset of women will be included in a participant survey at enrollment (during pregnancy) and after birth; we will measure satisfaction with care, locus of control, perceived social support, perceived stress, and depressive symptoms (see *Extended data*: File 3) ^18^. The postnatal questionnaire (unpiloted) will also include questions about health behaviors such as breastfeeding, post-partum family planning and use of an insecticide-treated bed net (see *Extended data*: File 4 and 5) ^18^.

Additionally, providers at both group ANC and standard ANC study sites will participate in a longitudinal survey, with questionnaires administered at baseline, nine, and 18 months (see *Extended data*: Files 6–8) ^18^. Measures of interest include level of education and years of clinical experience, job satisfaction and preferences, and perceived stress. We will report the uptake and effects of introducing urine pregnancy testing by CHWs and ultrasound by primary ANC providers.

### Data collection

All women enrolled in the study will be followed across pregnancy, birth, and up to 42 days after birth. We will leverage Rwanda’s existing data collection system to collect health outcomes data for the primary analysis. Specifically, we will use existing national data collection tools, including: ANC/PNC patient files; ANC, maternity, neonatal, and PNC registers; and the Rapid SMS database, a national real-time reporting and alert system which allows interactive communication between the CHW, health center, and the national centralized database, as our data sources. Given this, prior to the start of the RCT, the 36 health centers and the six district hospitals that receive referred clients from those health centers participated in data strengthening training to improve completeness, reliability and accuracy of existing data streams, especially registers and individual client files. First, the chief of nursing, the monitoring and evaluation officer and the data manager from each of the district hospitals were trained by data systems experts from the MOH, RBC and UR in a trainer-of-trainers model. Subsequently, those trained at the district level taught the same data strengthening module to data managers, CHW supervisors and ANC/PNC nurses at health centers with the support of project partners from RBC and the UR.

During the study duration, a PTBi—Rwanda data collector employed by this research team will be embedded at each study facility. These data collectors have at least an advanced diploma (A1) in general nursing or midwifery. The data collector introduces the study to each woman who presents for ANC and invites her to consent to data collection and analysis (see *Consent to participate* section below). At sites randomized to group ANC (Arms 3 and 4), the data collector assigns each enrolled woman to an antenatal group with her peers based on GA, communicates with ANC/PNC providers and CHWs to organize each health center’s group ANC schedule. However, these data collectors do not assist during group ANC or remind pregnant women to attend visits. In Arms 2 and 4, an ultrasound report form developed for this study will document examination findings, diagnosis, and examination process measures (see *Extended data*: File 10). Process measures of interest are the time spent on each ultrasound examination and the timing of the ultrasound (whether it was completed as part of a routine ANC visit or as a separate visit). At the community level, a UPT monthly form will be used to report on number of women tested, test results and referrals granted ( *see Extended data*: File 11) ^18^. These data will also be captured by the data collector in Research Electronic Data Capture version 2.35 (REDCap) ^23^.

Data elements are collected at enrollment (ANC 1 visit), during later ANC visits, delivery, and at PNC visits in the health center. In cases where a mother had a complication and was referred to the district hospital, usually the closest district hospital located within the catchment area, data collectors will find these data in the hospital registers. Data collectors are also responsible for monitoring the cohorts on a routine basis. This tracking process should alert staff when mothers do not attend ANC visits, give birth at a different facility, or do not attend PNC. Those who have not attended PNC by 12 weeks after the expected delivery date will be followed up by facility record review, telephone, or in immunization clinics to minimize loss to follow up. Table 3 displays the data collection strategy for each measure that will be analyzed and reported. Data collectors will also report adverse events and protocol violations (see *Extended data*: Files 1 and 2) ^18^.

**Table 3.** Outcome main indicators and data sources.

Outcome	Outcome indicator	Analysis Group	Source
**Gestational age**	GA (Recorded GA at birth VS. LMP- calculated GA)	All live births (Apgar > 0 at 1 min)	MAT Register
**28-day infant mortality among** **preterm neonates**	Preterm neonates dead at 28 days Preterm neonates alive at 28 days Preterm neonates lost to follow-up at 28 days	All live births (Apgar > 0 at 1 min) with GA < 37 weeks	MAT & NEO Register, RapidSMS
**42-day infant mortality among** **preterm neonates**	Preterm neonates alive at 42 days Preterm neonates dead at 42 days Preterm neonates lost to follow-up at 42 days	All live births (Apgar > 0 at 1 min) with GA < 37 weeks	PNC & Immunization Register, RapidSMS
**Adherence: four ANC visits**	Women who attended four ANC	All women who attended ANC 1	ANC Register
**Adherence: six-week PNC**	Women who attended PNC 1	All women who attended ANC 1	PNC Register
**ANC 1 within 1st trimester**	Women who attended ANC 1 <16 weeks	All women who attended ANC 1	ANC Register
**Identification of high risk**	Women with risk factors identified Number of women referred with risk factors	All women who attended ANC 1	ANC Register
**Maternal morbidity**	Cesaerean sections women referred for delivery women 'sick'	All deliveries	MAT Register, Referral Forms, RapidSMS

GA, gestational age; LMP, last menstrual period; MAT, maternity; NEO, neonatal; ANC, antenatal care; PNC, postnatal care;.

Other secondary outcomes will be measured. First, to survey a sub-set of participants across all trial arms, a convenience sample of the first five women to present for ANC per month are invited to participate in a baseline questionnaire measuring satisfaction with care, locus of control, perceived social support, perceived stress, and depressive symptoms. Similarly, among those who present at the health center with newborns approximately six weeks after birth, five women will be invited to participate in a follow-up questionnaire in which the same baseline questions are repeated and additional questions about postnatal health behaviors, including family planning uptake, insecticide-treated mosquito net use, and breastfeeding, are added *.* This will be an unmatched cohort of survey participants and the baseline and postnatal questionnaires (see *Extended data*: Files 3–5) ^18^. These questionnaires will be conducted in person by the data collector when the woman visits the health facility. Second, ANC and PNC providers in all arms are invited to participate in a longitudinal survey about job satisfaction, preferences, and perceived stress (baseline and follow-up questionnaires are available as *Extended data*: Files 6–8) ^18^. These data will be collected at study training meetings or administered by data collectors at the facilities. The baseline data of providers trained to deliver group ANC/PNC will be linked to their longitudinal scores of group ANC model fidelity assessed at regular intervals by the Master Trainers (assessment tool appears as *Extended data*: File 9) ^18^. Third, we will also conduct qualitative research among women and providers at nine and 18 months after implementation to inform program improvement. Four to six focus groups for women and four to six focus groups for providers will be convened to better understand their experiences of group ANC and PNC. More specifically, focus groups with providers will allow nurses and midwives to freely verbalized their ideas and concerns about group ANC and PNC, emphasizing on group care effect on their work, possible keys to success, effect on mothers and program changes or improvements. Focus groups with women will seek to capture reasons women will choose to attend or not group ANC and PNC, as well as soliciting suggestions to strengthen the program.

Data collectors abstract data from health center registers and patient files (and, where applicable, ultrasound reports and urine pregnancy test results) into REDCap loaded on password-protected tablet devices. Data will be synced weekly into the database via Wi-Fi network. The same devices will be used to record information from the participant and provider surveys and synced to the web-based application. Study personal identifiers will be separated from the rest of the data to ensure data protection and confidentiality. Access to the database will only be given to members of the study team. All personal information about enrolled participants will not be shared with any third party during and after the trial.

Focus group discussions will be audio recorded. To ensure confidentiality, each participant will be assigned a number; facilitators will refer to theses numbers when calling on participants during discussion. Audio files will be stored on password protected computer, only authorized staff will have access to the files. Audio recordings will be destroyed after transcription and analysis are complete.

### Sample size

Based on a review of RCT results included in the 2015 Cochrane Review of group versus conventional antenatal care for women ^24^, we assume the intraclass correlation coefficient (ICC) between Arms 1 and 2 and Arms 3 and 4 will be no larger than 0.01. We assume the standard deviation of GA at delivery is no larger than 4.3 weeks under both intervention and control arms. At 5% significance, with 36 facilities, this trial is powered (80%) to detect a 0.5-week difference in GA at delivery with 214 observations (ANC and outcome) per facility. Assuming a follow-up rate of 70%, 306 women per facility will be required. Just over 11,000 women will be recruited and we expect to follow almost 8,000 mother-newborn dyads through the period of the six-week PNC visit. At an average rate of 23 eligible recruits per month per facility, the total study duration will be (a) 14 recruitment months (from the end of May 2017 to August 2018, (b) an additional six to seven months to observe the outcomes of the last cohort of women recruited (February 2019), and (c) three additional months to complete data processing and analysis (May 2019).

### Statistical analyses

To compare the study groups, socio-demographic and reproductive health characteristics, and health care facility descriptive bivariate analyses stratified by study arm will be conducted using Chi-square and Student’s t-test statistics for categorical and continuous data, respectively. Similar unadjusted bivariate analyses will be presented for the primary and secondary outcomes. Unadjusted, intention-to-treat generalized estimating equations (GEE) linear regression with robust variance estimation analyses will be conducted to assess the effectiveness of the treatment regimen on the primary outcome variable, gestation at delivery, and analogous linear and logistic analyses of will be conducted to assess continuous and categorical secondary outcomes (Model 1). For these outcomes, similar analyses will be conducted to control for design effect (Model 2) and the effects of any additional unforeseen differences in measured study group characteristics (Model 3). Model 3 will include all covariates that are statistically significant and judged as important differences between the study group (e.g. not simply significantly different due to large sample size) as identified by a backward stepwise Wald analysis (Model 3), which removes the covariate least associated with the outcome, and continues in that manner to remove covariates not significantly associated with the outcome at the α=0.05 level.

Model 1: Outcome=Independent Causal Variable
Model 2: Outcome=Independent Causal Variable, adjusted for design effect
Model 3: Outcome=Independent Causal Variable, adjusted for design effect plus (retained) covariates

Additionally, we propose comparing these methods with data-adaptive targeted maximum likelihood estimators (TMLE) ^25^. These would attempt to leverage the hierarchical structure of the data and would adjust for covariates through a data-adaptive algorithm. Multi-level analyses to adjust for facility-level characteristics will be considered.

The main analyses will compare the control group (Arms 1+2) with the intervention group (Arms 3+4). Subanalyses by study arm will be conducted.

Arm 1: Standard ANC/PNC care
Arm 2: Standard ANC/PNC care, with early pregnancy testing and ultrasound
Arm 3: Group ANC/PNC only
Arm 4: Group ANC/PNC care, with early pregnancy testing and ultrasound

All descriptive and statistical analyses, including survey data, will be performed using SPSS for Windows version 23, Stata SE version 15.1 and R version 3.5 ^26, 27^. Qualitative data from focus group discussions at nine and 18 months after implementation will be audio recorded, transcribed and translated from Kinyarwanda to English.. Transcripts will be organized into thematic areas with Atlas ti 7.5.18 using a content analysis approach.

### Trial monitoring

The trial will be monitored by a five-member independent Data and Safety Monitoring Board (DSMB). DSMB members include two obstetrician-gynecologists, one pediatrician, one midwife, and one biostatistician; two of these members are Rwandan and all are East African. The DSMB charter is available as *Extended data*: File 14 ^18^.

The DSMB will convene after trial initiation, with subsequent meetings at least every six months and will perform up to two interim data analyses. The DSMB has agreed on the Haybittle–Peto rule ^28, 29^ as the stopping boundary to be used for interim analyses. In the case that application of the Haybittle-Peto rule results in a recommendation to terminate the trial, the DSMB will consult with the study investigators and other partners to make a final decision. Adverse events will be reported immediately to the ethical review boards and will be summarized and presented to the DSMB at each of their meetings.

### Ethics approval

This study protocol was reviewed and approved by the Rwanda National Ethics Committee (No 0034/RNEC/2017) and the UCSF Institutional Review Board (No 16-21177). A waiver of parental consent for any adolescent 15 years of age or older was granted by the Rwanda National Ethics Committee, allowing adolescents over 15 to consent to participation in primary and secondary interventions and data collection and analysis.

All members of the research team were trained in ethical practices in human research. Research staff will emphasize that participation in the study is voluntary and that refusal to participate in the study will not results in negative repercussions. If any modifications to the study protocol are made, these modifications will be submitted to both ethical review boards for approval. This study was registered on ClinicalTrials.gov, ID NCT03154177 on May 16, 2017.

### Consent to participate

Prior to enrollment or interview, data collectors will administer consent to participants. Each participant will be given the opportunity to read a written consent form or if illiterate have it read to her/him by a witness *(see Extended data*: Files 12 and 13f) ^18^. The consent statement will explain the study objectives, requirements, potential risks, privacy and ethical obligations of the research team. The participant will complete the consent process by agreeing or disagreeing to the consent statement. The consent form will be administered in Kinyarwanda (the local language) and only participants who agree to participate and sign the written consent form will participate.

### Dissemination of data and materials

Final datasets will be jointly owned by all investigators. As per our funder’s open access policy, de-identified datasets and statistical code will be made publicly available on request once we have published on our primary outcomes.

Results will be disseminated in a national dissemination meeting, at international conferences and through publication. No specific efforts will be made to reach participants.

### Study status

Enrollment and data collection started on May 25, 2017 and ended on December 31, 2018. Data processing and analysis are currently being conducted.

## Discussion

In WHO’s 2016 *Recommendations on antenatal care for a positive pregnancy experience*, the authors write that

“communication and support functions of ANC are key, not only to saving lives, but to improving lives, health-care utilization and quality of care. Women’s positive experiences during ANC and childbirth can create the foundations for healthy motherhood.” ^16^

Group ANC seems to hold the promise of improved effectiveness of health message communication in a peer-to-peer education model, enhanced support and cohesion among pregnant women in a community, and increased satisfaction with and uptake of ANC.

This trial will provide much-needed evidence to advance the field. First, it will examine the health effects of group ANC in the context of low- and middle-income countries (LMICs) and within an intent-to-treat approach almost identical to routine health service delivery in that context. Second, it is the first to test group care as applied to the four-visit Focused ANC model widely used in LMICs, and thus will provide evidence about whether this innovative model can improve adherence to the four-visit schedule. Third, the trial will also report whether the group care model impacts attendance at six-week PNC in a context in which PNC attendance is currently very low. Fourth, the trial will also provide feasibility evidence regarding the use of CHWs as co-facilitators in group care. Lastly, this trial further provides additional insight into the relative effects of basic obstetric ultrasound at health centers and urine pregnancy testing at the community level on early uptake of ANC and adherence to the recommended visit schedule.

This is the largest trial of group ANC to date, powered to detect a difference in GA at birth. The implementation of group ANC at this scale within the practical realities of a national maternity care system will inform global stakeholders as they make decisions at the policy, system, facility, and individual levels about the optimal ANC service model for the mothers and newborns they serve.

## Data availability

### Underlying data

All data underlying the results are available as part of the article and no additional source data are required.

### Extended data

Open Science Framework: Group antenatal care versus standard antenatal care and effect on mean gestational age at birth in Rwanda: a cluster rancdomized trial. https://doi.org/10.17605/OSF.IO/9CQEZ ^18^.

This project contains the following extended data:

– File 1 Adverse event form.docx
– File 2 Prococol violation form.docx
– File 3 Baseline participant questionnaire.docx
– File 4 Postnatal participant questionnaire_standard .docx
– File 5 Postnatal participant questionnaire_group.docx
– File 6 Baseline provider questionnaire.docx
– File 7 Follow uo provider questionnaire_standard.docx
– File 8 Follow uo provider questionnaire_group.docx
– File 9 Model fidelity assessment.docx
– File 10 Ultrasound report.pdf
– File 11 UPT referral form.pdf
– File 12a Group care Participant Consent Form.docx
– File12b Standard care Participant Consent Form.docx
– File 12c Standard care Participant Assent Form - Adolescents.docx
– File 12d Standard care Parental permission form
– File 12e Group care Participant Assent Form - Adolescents.docx
– File 12f Group ANC Parental permission form.docx
– File 13a Group care Participant Consent Form_KIN.docx
– File13b Standard ANC Participant Consent Form_KIN.docx
– File 13c Standard Care Participant Assent Form - Adolescents_KIN.docx
– File 13d Standard ANC Parental permission form_KIN.docx
– File 13e Group Care Participant Assent Form - Adolescents_KIN.docx
– File 13f Group ANC Parental permission form_KIN.docx
– File 14 PTBi Rwanda DSMB charter
– File 15 List of Health centers

Extended data are available under the terms of the Creative Commons Zero "No rights reserved" data waiver (CC0 1.0 Public domain dedication).

### Reporting guidelines

Open Science Framework: SPIRIT checklist for ‘Group antenatal care versus standard antenatal care and effect on mean gestational age at birth in Rwanda: a cluster randomized controlled trial’. https://doi.org/10.17605/OSF.IO/9CQEZ

Data are available under the terms of the Creative Commons Zero "No rights reserved" data waiver (CC0 1.0 Public domain dedication).
