# Supplementary material for: Group antenatal care versus standard antenatal care and effect on mean gestational age at birth in Rwanda: protocol for a cluster randomized controlled trial
Source: Gates Open Res. 2019 Sep 27;3:1548. [Version 1] doi: 10.12688/gatesopenres.13053.1 (PMC6792348; doi:10.12688/gatesopenres.13053.1)
Supplement: Peer review history

# Peer review: Reviewer response for version 1

**DOI:** 10.21956/gatesopenres.14181.r27955

**Published:** 2019-10-14

**Authors:** Jody Lori

**Affiliations:** 1 Department of Research, Office of Global Affairs and Pan American Health Organization/ World Health Organization Collaborating Center, University of Michigan School of Nursing, Ann Arbor, MI, USA

Thank you for the opportunity to review the manuscript “Group antenatal care versus standard antenatal care and effect on mean gestational age at birth in Rwanda: protocol for a cluster randomized controlled trial” by Musange, SF *et al.* The manuscript describes the research protocol for a pair-matched cluster randomized controlled trial with four arms. This manuscript is well-written and clearly describes the protocol used to conduct the above trial. This detailed protocol will add to the literature on approaches to study group antenatal and postnatal care in low resource settings. It is the largest cluster RCT of group ANC and PNC ever conducted. The authors present extensive background and rationale for the study and clearly describe the intended evaluation including primary and secondary outcomes. 

 The methods section provides appropriate detail on the study design and setting including power calculations and rationale. The primary and secondary outcomes are clear. Data collection and data quality assurance are clearly presented. All tables and figures are clear and do not repeat information provided in the text. Supplementary files provide additional details for ease in replication of the study. Statistical analysis for both quantitative and qualitative results is appropriate. No results or conclusions are reported as this manuscript is a descriptive study protocol.

 It is not clear from the manuscript if ultrasound is unavailable to study participants currently or if women in control groups (without U/S) will have the opportunity to receive U/S during their pregnancy through other mechanisms not associated with this protocol. The authors also state that the data collectors do not remind pregnant women to attend visits but they do not say if nurses/midwives (or anyone else) is reminding women of visits in any of the four study arms. It would help the reader to understand these details. I am not familiar with the Rwanda context but women in other parts of Africa often travel to live with family members for delivery and remain there for varying lengths of time postpartum. While a plan for following women who deliver at the district hospital is described, additional information on the tracking of women who travel out of the area for delivery is less clear.

 Overall, this is a clear and precise manuscript outlining the details of a large cluster RCT of group antenatal and postpartum care in a low-resource setting. I commend the authors for their detailed description of each step of the study allowing replication by other researchers and look forward to reading the results from their study.

I confirm that I have read this submission and believe that I have an appropriate level of expertise to confirm that it is of an acceptable scientific standard.

# Peer review: Reviewer response for version 1

**DOI:** 10.21956/gatesopenres.14181.r27956

**Published:** 2019-10-11

**Authors:** Mark M. Kabue

**Affiliations:** 1 Johns Hopkins University, Baltimore, MD, USA

**Summary:**

 The manuscript titled “Group antenatal care versus standard antenatal care and effect on mean gestational age at birth in Rwanda: protocol for a cluster randomized controlled trial”, describes the design and intended evaluation of a four-arm cluster RCT of a combined group ANC/PNC model compared to routine antenatal care. Overall, the manuscript is well-written and clearly describes the protocol used to conduct the trial. The results will add to the growing body of knowledge, adding on to findings of cRCT on group ANC done in Kenya and Nigeria thus providing additional evidence on the subject matter especially in low resource settings. The study is anchored within the context of the *WHO Recommendations on Antenatal Care for a Positive Pregnancy Experience.*

 The manuscript provides an appropriate level of detail on the study design and setting including power calculations and rationale. The primary and secondary outcomes are clear. The supplementary files provide additional details on the type of data collected to answer the research questions. Statistical analysis is described well. No results are reported since this is a study protocol manuscript

**Specific comments by section:** 

**1) Abstract: **Well written, concise.

**2) Background: **Good literature review, citing available literature on the subject matter especially from low resource settings like Ghana. Include the findings of cRCT done in Nigeria and Kenya ^1^ as well along with any other relevant studies from other parts of the world on group care.

**3) Methods:** Study design is well articulated to answer both the primary and secondary research questions. 

- Under study implementation, it is stated that providers will determine the women’s' due date “based on best obstetric estimate”. Since various methods are available for determining the EDD, provide more details on how this process was standardized. Also explain how the final EDD was arrived at when there was conflict between various methods especially in the absence of Ultrasound.
- “in addition, postnatal measurements of every newborn will be used to estimate GA at birth and assign a corrected GA if needed”. Clarify whether this does not present “potential bias” if the GA - the primary outcome - is adjusted AFTER the baby is born especially in the intervention arm since the providers are not “blinded” with respect to the study arm.
- Under data collection, clarify whether locater information had been collected from all women during enrolment in order to enable the CHWs to trace them. What information was used to trace the women?
- With regard to measuring satisfaction, provide a rationale for sampling only a few women and using “convince sampling”. Potentially very important data on experience of care can be collected through this questionnaire. Consider using a larger sample size of women selected in a more systematic manner.
- The data sources are appropriate bearing in mind that the findings will inform the roll-out of group antenatal care within the existing health systems, thus using the national data collection systems.

**4) Discussion: **Brief and well written, framing the importance of the trial and its potential contribution.

 I look forward to seeing the findings of this trial. The study will make a major contribution to the literature on group antenatal care.

I confirm that I have read this submission and believe that I have an appropriate level of expertise to confirm that it is of an acceptable scientific standard.

## References

[1] : Impact of group antenatal care (G-ANC) versus individual antenatal care (ANC) on quality of care, ANC attendance and facility-based delivery: A pragmatic cluster-randomized controlled trial in Kenya and Nigeria. *PLoS One*.2019;14(10) : 10.1371/journal.pone.0222177 e0222177 10.1371/journal.pone.0222177 31577797PMC6774470
